# Comparative computational analysis to distinguish mesenchymal stem cells from fibroblasts

**DOI:** 10.3389/fimmu.2023.1270493

**Published:** 2023-09-26

**Authors:** Bettina Budeus, Kristian Unger, Julia Hess, Hanna Sentek, Diana Klein

**Affiliations:** ^1^ Institute for Cell Biology (Cancer Research), University Hospital Essen, University of Duisburg-Essen, Essen, Germany; ^2^ Research Unit Radiation Cytogenetics, Helmholtz Zentrum München, German Research Center for Environmental Health GmbH, Neuherberg, Germany; ^3^ Clinical Cooperation Group Personalized Radiotherapy in Head and Neck Cancer, Helmholtz Zentrum München, German Research Center for Environmental Health GmbH, Neuherberg, Germany

**Keywords:** adult stem cells, mesenchymal, cellular identity, hox code, signaling pathways, inflammation

## Abstract

**Introduction:**

Mesenchymal stem cells (MSCs) are considered to be the most promising stem cell type for cell-based therapies in regenerative medicine. Based on their potential to home to diseased body sites following a therapeutically application, these cells could (i) differentiate then into organ-specific cell types to locally restore injured cells or, most prominently, (ii) foster tissue regeneration including immune modulations more indirectly by secretion of protective growth factors and cytokines. As tissue-resident stem cells of mesenchymal origin, these cells are morphologically and even molecularly- at least concerning the classical marker genes- indistinguishable from similar lineage cells, particularly fibroblasts.

**Methods:**

Here we used microarray-based gene expression and global DNA methylation analyses as well as accompanying computational tools in order to specify differences between MSCs and fibroblasts, to further unravel potential identity genes and to highlight MSC signaling pathways with regard to their trophic and immunosuppressive action.

**Results:**

We identified 1352 differentially expressed genes, of which in the MSCs there is a strong signature for e.g., KRAS signaling, known to play essential role in stemness maintenance, regulation of coagulation and complement being decisive for resolving inflammatory processes, as well as of wound healing particularly important for their regenerative capacity. Genes upregulated in fibroblasts addressed predominately transcription and biosynthetic processes and mapped morphological features of the tissue. Concerning the cellular identity, we specified the already known HOX code for MSCs, established a potential HOX code for fibroblasts, and linked certain HOX genes to functional cell-type-specific properties. Accompanied methylation profiles revealed numerous regions, especially in HOX genes, being differentially methylated, which might provide additional biomarker potential.

**Discussion:**

Conclusively, transcriptomic together with epigenetic signatures can be successfully be used for the definition (cellular identity) of MSCs versus fibroblasts as well as for the determination of the superior functional properties of MSCs, such as their immunomodulatory potential.

## 1 Introduction

Mesenchymal stem cells (MSCs) comprise a heterogeneous cell population of mesodermal origin with capabilities for multipotency and self-renewal. Initially described as ‘fibroblast precursors’ by Friedenstein et al. (1, 2), MSCs have become one of the most frequently investigated stem cell types 50 years later and are supposed to be the most promising stem cell type for cell-based therapies (3–6). As tissue-resident stem cells, MSCs are important orchestrators of tissue homeostasis, contributing to the maintenance of organ integrity by regulating the tissue’s turnover, renewal, and repair. MSCs have an intrinsic ability to migrate to the site of tissue damage and can then actively promote tissue regeneration by differentiating into specific cell types to locally restore tissue injury. However, it has been shown that, in contrast to this direct action, that MSCs exert a predominantly indirect effect through paracrine secretion of bioactive molecules capable of stimulating the recovery of injured cells and limiting inflammation (7–9). In addition, MSCs lack immunogenicity and can be considered safe for clinical applications (10, 11). These unique properties have promoted numerous applications of MSCs.

During ontogeny, these MSCs and MSC-like progenitor cell populations, preferentially derived from the somatic lateral plate mesoderm, are particularly involved in the development of mesenchyme-derived evolving structures and organs (12). In the adult organism, the bone marrow remains the classic known reservoir for MSCs, whereby MSCs can be found in and effectively isolated from almost every organ, although it remains to be finally clarified if MSCs are developmentally determined tissue-resident cells or are continuously replenished by the bone marrow (6). The omnipresence of putative MSCs and thus the distribution throughout the body is explained by their localization close to blood vessels (13, 14), whereby the frequently used term ‘perivascular’ localization correctly indicates vessel association but remains not anatomically correct (15, 16). The vascular stem cell niche where different stem and progenitor cells including MSCs reside, also termed the ‘vasculogenic zone’, is located within the vascular wall of (larger) blood vessels close to the smooth muscle cell layer (tunica media), particularly within the vascular adventitia, representing the interface between the vessel wall and surrounding tissue that is the perivascular space (14, 15, 17–20). With regard to the therapeutic potential, it can be assumed that these vascular wall-derived MSCs (VW-MSCs) might be particularly well suited to exhibiting superior repair capabilities for the protection and curative treatment of vascular diseases because of their tissue-specific action (9, 19, 21).

MSCs are classically characterized by the ability to adhere to plastic; the expression of an MSC surface marker panel including CD90, CD73, and CD105, while lacking CD11b, CD79a, CD19, and HLA-DR; and the potential to differentiate into mesodermal tissues (22, 23). These conventional MSC properties turned out to be unspecific for phenotypic characterization of MSCs compared to other mesodermal cells, particularly fibroblasts, indicating that there is an urgent need to identify additional cell type-specific markers (24). The appearance of both cell types -at least at first glance- concerning morphology (‘a fibroblast-like morphology’) and phenotype (expression of certain cell surface markers, plastic adherence, and rigorous *in vitro* expansion) is so similar that MSCs seem to be indistinguishable from fibroblasts (21, 25). That may be based, at least partially, on their similar developmental origin, as the majority of fibroblasts in the body derive from precursors of paraxial mesoderm and lateral plate mesoderm (26). Here, we investigated global gene expression and methylation profiling and applied accompanying computational tools in order to specify differences between MSCs and fibroblasts, to further unravel potential identity genes, and to highlight MSC signaling pathways with regard to their trophic and immunosuppressive action, which is a prerequisite for the clinical application of MSC-based therapies.

## 2 Materials and methods

### 2.1 Microarray-based gene expression analysis

Primary adventitial MSCs (VW-MSCs) were routinely isolated from human internal thoracic arteries obtained during surgery (fragments) as described previously (21, 27, 28) and cultivated on plastic cell culture plates using complete human MSC-GM media (PromoCell, Heidelberg, Germany). Primary fibroblasts derived from healthy donors were cultivated in fibroblast medium (DMEM high glucose, 10% FCS, 50 U/ml Pen/Strep, 1% sodium pyruvate, 1% glutamine, 1% non-essential amino acids, and 0.2% β-mercaptoethanol) (21). All experiments were performed in strict accordance with local ethical and biohazard regulations and were approved by the local ethics committee. Informed consent (written form, Nr.10-4363 and 17-7454-BO) was obtained from the Ethik-Kommission, University Medical Faculty, Essen, Germany (16, 27, 29, 30). Total RNA was isolated from VW-MSCs and fibroblasts (n=4 per group) and global gene expression profiling was performed as previously described using SurePrint G3 Human Gene Expression 8x60k microarrays (AMADID 028005, Agilent Technologies) according to the manufacturer’s protocol (21). Data quality assessment, preprocessing, normalization, and differential expression analyses were conducted using the R Bioconductor packages limma (31) as previously described (21).

Heatmaps with unsupervised hierarchical clustering were generated based on the gene expression z-scores using the ComplexHeatmap R-package (32) and standard settings. Gene Set Enrichment Analysis was performed with the R-package fgsea (33) and gene sets from v2023.1 were downloaded from the Molecular Signatures Database (34, 35) as well as custom gene sets as indicated. Gene association analysis of a specific gene was based on gene sets from the Molecular Signatures Database. Volcano plots were generated with the R-package EnhancedVolcano (https://github.com/kevinblighe/EnhancedVolcano). Real-time RT-PCR quantifications following cDNA synthesis using QuantiTect Reverse Transcription (Qiagen, Hilden, Germany) were performed according to the manufacturer’s instructions and were carried out using specific deoxy-oligonucleotide primers as previously described (21, 27). Wilcoxon rank-sum test was used to analyze the statistical difference between MSCs and FIBs regarding gene expression from normalized microarray data or normalized qPCR data.

### 2.2 Global DNA methylation analysis

Processing of DNA methylation arrays was performed on an Illumina (San Diego, CA, USA) platform as previously described (21). Total gDNA was isolated from primary fibroblasts (n=5) and VW-MSCs (n=4). Bisulfite conversion of 500ng of DNA was done using the EZ DNA Methylation Kit (Zymo Research, Orange, CA, USA) as described before (21). Converted gDNA was processed using Infinium®MethylationEPIC BeadChips (Illumina, San Diego, CA, USA) following the Illumina Infinium HD Methylation instructions. GenomeStudio (version 2011.1) with the Methylation Module (version 1.9.0) was used to process the raw image data generated by the BeadArray Reader. Initial quality control of assay performance was undertaken using the “Control Dashboard” provided by GenomeStudio Software, including the assessment of staining, extension, hybridization, target removal, bisulfite conversion, specificity negative, and non-polymorphic control and checking for number of detected CpG sites. Methylation beta values were processed using the R packages limma (31), minfi (35), missMethyl (36), and DMRcate (37). The R-package Gviz was used to visualize the single CpG methylations, regions, and genes (38), and the R package, trackviewer (39), was used for the display of specific beta values in single HOX genes. Common methylation alterations were assessed following the grouping of CpGs by the indicated region and/or gene and relative to the genomic location (transcription start site 1500, TSS1500: 200–1500 bp upstream of the TSS), TSS200: 0–200 bp upstream of the TSS, 5′ untranslated region (5′UTR), 1st exon, gene body, and 3′UTR). The annotation of specific genetic regions was done with IlluminaHumanMethylationEPICanno.ilm10b4.hg19.

## 3 Results

### 3.1 Gene expression profiling of MSCs versus fibroblasts

**Figure 1:**
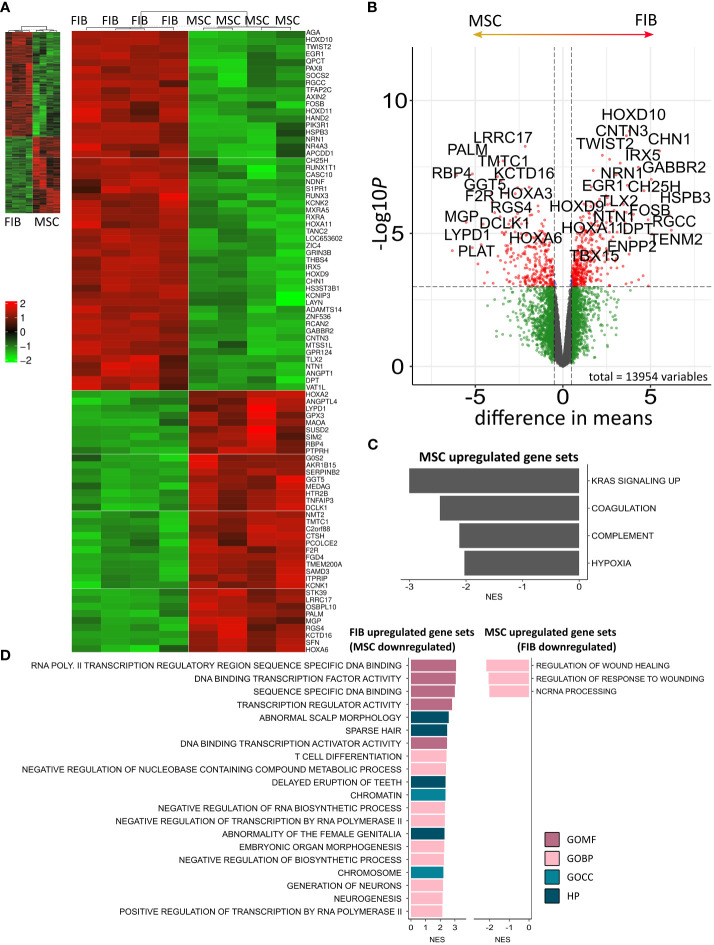
Global gene expression analysis to determine differences between MSCs and FIB. **(A)** Hierarchical clustering heatmap of genes with log-fold change > 0.5 and p-value < 0.001 (small heatmap) and of genes with log-fold change > 1 and p-value < 0.001 (large heatmap) of primary vascular wall-derived MSCs and primary dermal fibroblasts as determined by microarray analysis. **(B)** Volcanoplot with all genes. Significant differences in 1352 transcripts (foldchange > 0.05 and adjusted p-value cut-off of 0.001) are highlighted in red. Genes upregulated in MSC are on the left, genes upregulated in FIB are on the right site. Top 25 genes are named. **(C)** Significantly enriched Hallmark and **(D)** significantly enriched C5 ontology gene sets showing normalized enrichment score (NES) for significantly upregulated and downregulated gene sets. BP, biological process; CC, cellular component; MF, molecular function; HP human phenotype. Biological replicates as indicated: MSC n=4; FIB n=4). Statistics: limmas moderated t-test, adjusted by “BH”).

**Figure 2:**
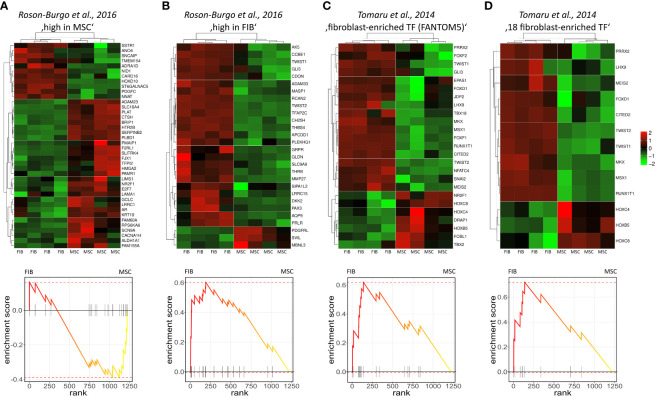
Gene set enrichment analysis using gene sets specific for different MSCs and FIB. Genes highly expressed in human MSCs derived from placental tissue **(A)** as compared to human skin fibroblasts **(B)** were used (40). Fibroblast-enriched candidate factors derived from the gene regulatory interactions analyses in fibroblasts by the FANTOM5 consortium **(C)** and further specified eighteen fibroblast-enriched transcription factors **(D)** as potential cell identity genes maintaining the fibroblast state were used (41). Displayed are the heatmaps (top panels) and enrichment plots (lower panels) of significantly enriched genes.

**Figure 3:**
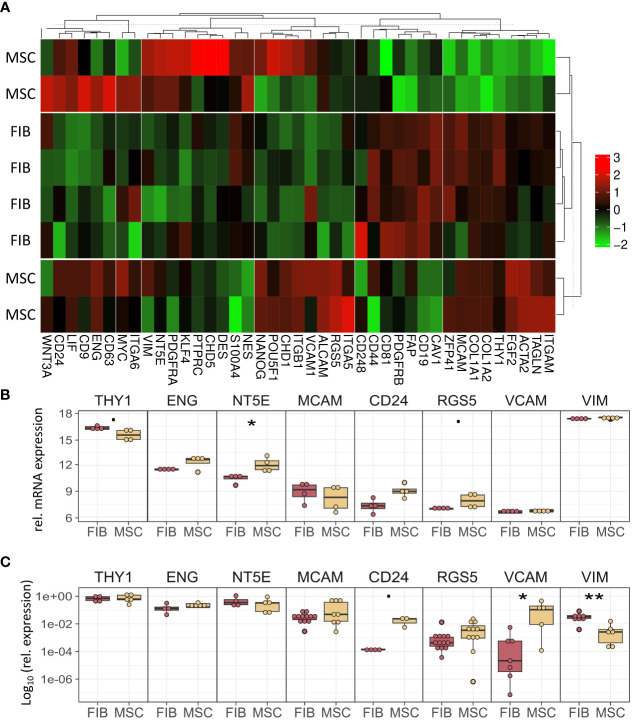
MSCs and FIB express the same cell phenotypic marker genes that are known to be expressed in mesodermal cells. **(A)** Heatmap of indicated classical marker genes for MSCs and FIB. **(B)** Relative mRNA expression levels obtained from the microarray expression profiles of eight selected signature genes are separately depicted. **(C)** Respective transcript levels were further quantified using Real-Time RT-PCR and are shown as relative expression to beta-actin (log_10_ transformed). Biological replicates as indicated: MSC n=4; FIB n=4; statistical analysis: Wilcoxon rank-sum test,.=p<0.1, *p<0.05, **p<0.01.

The molecular profiles of primary human vascular wall-derived MSCs (MSCs) and primary dermal fibroblasts (FIB) were characterized and compared by microarray gene expression profiling (**Figure 1**). A hierarchical clustering was built based on expression levels of genes (with log-fold change >0.5 or >1 and p-value <0.001) identifying signatures that validated at the transcriptome level the distinct natures of MSCs and FIB (**Figure 1A**). The volcano plot analysis further highlighted differential gene expressions in both cell types (**Figure 1B**). Using an adjusted p-value cut-off of 0.05, 1352 transcripts were found to be differentially expressed in both cell types with 565 genes found to be upregulated in MSCs as well as 787 upregulated genes in FIB (**Figure 1A**: small heatmap and **Figure 1B**). Gene set enrichment analysis (GSEA) was performed according to the Hallmark gene set (including 50 gene sets of specific well-defined biological states or processes) (**Figure 1C**) and the C5 ontology gene sets (including 15937 gene sets containing biological process, cellular component, molecular function, and human phenotype components) (**Figure 1D**) from the molecular signatures database MSigDBv6.1 (Broad Institute). GSEA revealed a strong signature for KRAS signaling, known to play an essential role in stemness maintenance and regulation of coagulation and complement, which is decisive for resolving inflammatory processes, and in wound healing, particularly important for the regenerative capacity in MSCs. Gene sets upregulated in fibroblasts predominately addressed transcription-related and biosynthetic processes, and to a lesser extent, expression of transcripts related to tissue morphological features (**Figure 1D**). To further confirm that MSCs and FIB displayed a gene expression signature corresponding to classical and well-known MSC and FIB phenotypes reported by others, we performed GSEA again (**Figure 2**). Using gene sets specific for MSCs derived from the human placenta as compared to primary skin fibroblasts (40), we confirmed that the vascular wall-derived MSCs closely resembled the transcriptional signature of these MSCs while the FIB displayed the typical signature of (skin) fibroblasts (**Figures 2A, B**). In previous studies, we already confirmed the MSC phenotype of our MSCs using Rohart MSC signature genes (21, 42). Further analyses of gene regulatory interactions in fibroblasts as identified by the FANTOM5 consortium using a fibroblast-enriched candidate factor as well as certain fibroblast-enriched transcription factors (41, 43) again validated the fibroblast phenotype of FIB and thus confirmed discrimination from MSCs by transcriptome profiling. In contrast, classical mesodermal and stem cell- and fibroblast-related marker genes including CD73/NT5E, CD90/THY1, CD105/ENG, CD146/MCAM, and CD24 could not – as already known- be used to distinguish both cell types (**Figure 3A**). Notably, MSCs tend to have higher expression levels of CD146/MCAM, CD24, and VCAM1, while increased expression levels of VIM could be estimated for FIB (**Figures 3B, C**).

### 3.2 The potential ‘HOX code’ of FIB

**Figure 4:**
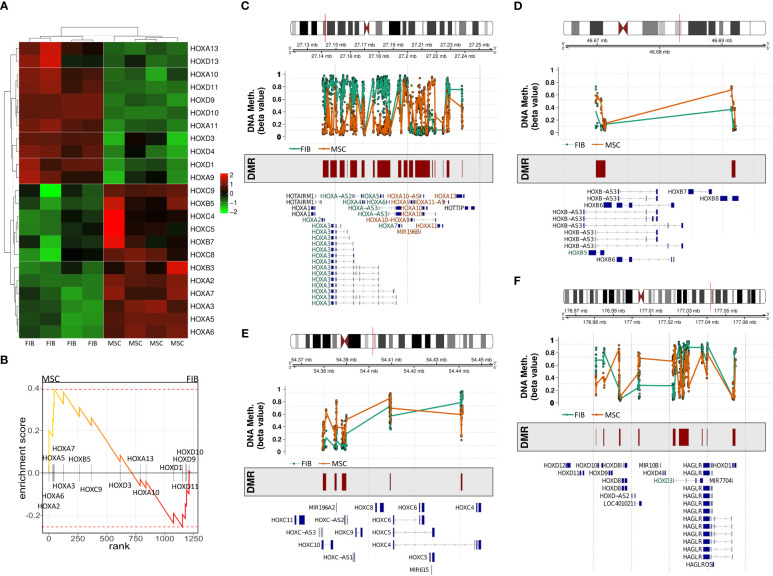
Expression and methylation of HOX genes in MSCs and FIB as estimated by global gene profiling. **(A)** Heatmap of the relative mRNA expression levels of indicated HOX genes (23 of 39 HOX genes) for MSC and FIB as obtained from the microarray expression profiles. **(B)** GSEA plot of the obtained HOX gene set. DNA methylation of the HOX gene regions for MSCs (colored in orange) and FIB (colored in brown) were summarized for the **(C)** HOXA, **(D)** HOXB, **(E)** HOXC, and **(F)** HOXD gene region. Differentially methylated regions (DMR) and gene annotations are additionally shown.

**Figure 5:**
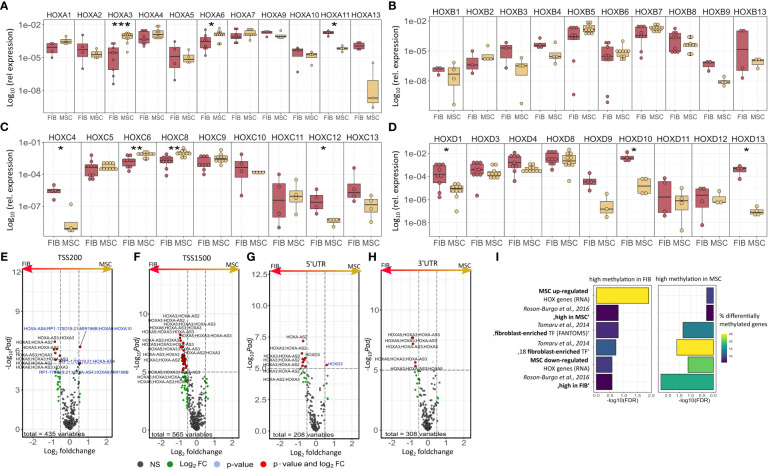
Expression levels of the 39 human HOX genes in MSCs compared to FIB as quantified by Real-Time RT-PCR for **(A)** HOXA, **(B)** HOXB, **(C)** HOXC, and **(D)** HOXD genes. Respective transcript levels are shown as relative expression to beta-actin (log_10_ transformed). Biological replicates as indicated: individual symbols depict different biological replicates (with a minimum of n=4); statistical analysis: Wilcoxon rank-sum test,.=p<0.1, *p<0.05, **p<0.01. Volcano plots of differentially methylated HOX regions assigned to **(E)** TSS200, **(F)** TSS1500, **(G)** 3’UTR, and **(H)** 5’UTR. Regions up-regulated in MSC are on the left and colored in blue (high methylation values), regions up-regulated in Fib are on the right and colored in black. **(I)** GSEA of differentially methylated regions of important MSC/Fib genesets from **Figure 2**. Statistical analysis: Wilcoxon rank-sum test,.=p<0.1, *p<0.05, **p<0.01, ***p<0.005. for RT-PCR data and limmas moderated t-test, adjusted by “BH” for chip data.

**Figure 6:**
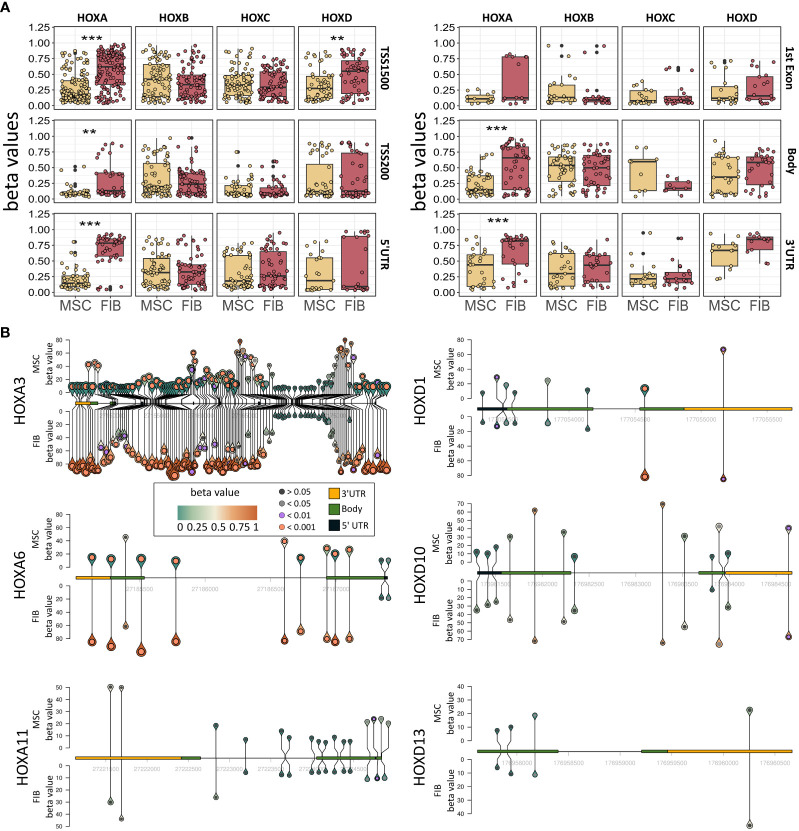
Epigenetic analysis of HOX gene cluster regions and potential HOX code candidates. **(A)** Methylation pattern of HOXA, HOXB, HOXC, and HOXD gene regions. Beta values for methylation are shown for FIB and MSCs for all CpGs in TSS1500, TSS200, 5’UTR, 1st Exon, Body, and 3’UTR. Annotation according to IlluminaHumanMethylationEPICanno.ilm10b4.hg19 Statistical analysis: Wilcoxon rank-sum test, **p<0.01, ***p<0.001. **(B)** Methylation pattern of HOXA3, HOXA6, HOXA11, HOXD1, HOXD10 und HOXD13. For each gene, the methylation of each measured CpG site is shown with the height and color of the pins representing the beta value of the CpG site. The circle inside the pin shows if there is a significant difference between FIB and MSCs at this position (black: no significant difference, grey: adj. p < 0.05, lilac: adj. p < 0.01 and orange: adj. p < 0.001, limmas moderated t-test, adjusted by “BH”). The size of the pins indicates the absolute fold change between MSCs and FIB (no scale given, small is low FC, large is high FC). The gene itself is shown from 5’ UTR (yellow) to 3’UTR (black) with gene body (green) only shown as exons. Intronic sequences are not shown as boxes.

**Figure 7:**
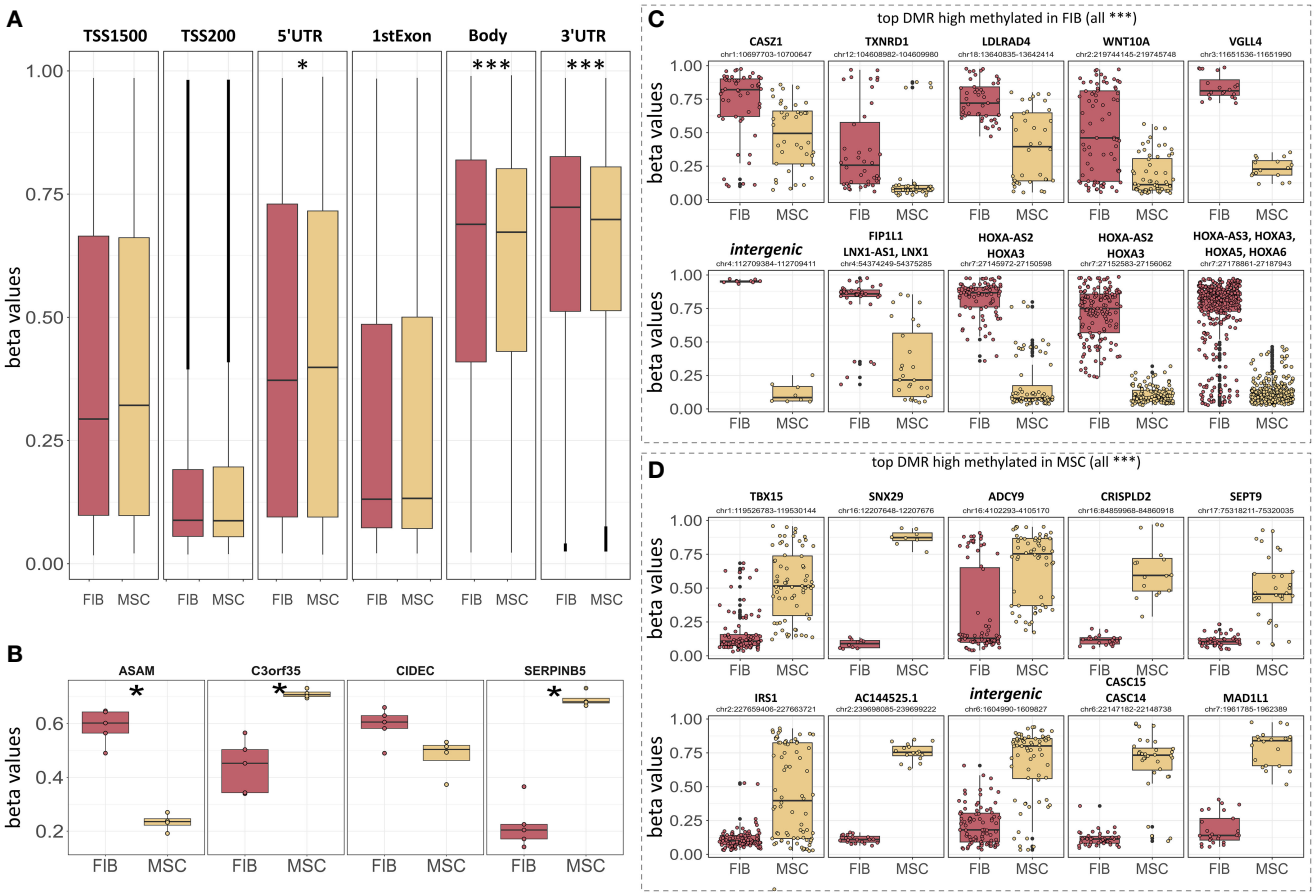
Epigenetic classification of MSCs and fibroblasts. **(A)** Overall DNA methylation levels of CpGs assigned to the indicated region categories. Regions were identified using IlluminaHumanMethylationEPICanno.ilm10b4.hg19. **(B)** DNA methylation levels of the four indicated CpGs known as the ‘Epi-MSC Score’ (45) successfully discriminating MSCs from FIB are shown. The 10 most significantly different differentially methylated regions (DMR) that were highly methylated in FIB **(C)** and MSCs **(D)** are additionally shown. Statistical analysis: Wilcoxon rank-sum test: *p<0.05, **p<0.01, ***p<0.005.

The observation that single HOX transcription factors could be found within the most differentially expressed genes in MSCs compared to FIB (**Figures 1A, B**), together with the already established HOX code as an integral regulator of (stem) cell identity and cell fate for MSCs particularly for VW-MSCs (16, 21, 27, 44), encouraged us to designate a HOX-specific-expression pattern and thus establishing a HOX code for fibroblasts (**Figure 4**). Herein, transcript levels of the HOX candidates as detected by the microarray analysis (23 HOX genes) revealed obvious differences between both mesodermal cell types (**Figures 4A, B**). Whereas MSCs were characterized by the expression of more central HOX genes of the HOX clusters HOXA, HOXB, and HOXC, the HOX-expressing patterns of FIB comprise predominantly posterior HOX genes of the HOXA and HOXD clusters. In parallel, we investigated DNA methylation of the HOX gene regions obtained from global DNA methylation profiles, comparing MSCs with FIB (**Figures 4C–F**). Consistent with the gene expression analysis, the earlier HOXA cluster genes (HOXA1-HOXA3) were highly methylated in FIB in contrast to the lower methylated regions comprising posterior HOXA (HOXA9-HOXA13) genes. This pattern was found in reverse order in MSCs, particularly for HOXA3 (**Figure 4C**). Also, the lncRNA HOTAIRM1, known to be involved in transcriptional regulation of the HOXA genes, turned out to be highly methylated in FIB. Likewise, HOXA antisense RNA HOXA-AS2 within the anterior HOXA region was highly methylated in FIB whereas the microRNA MIR-196b located in the posterior HOXA region was highly methylated in MSCs. Concerning the HOXB and HOXC regions, reduced methylation sites and partial values at all were observed in both cell types with increased methylation values of HOXB antisense RNA HOXB-AS3 in MSCs and of HOXB8, as well as (again) higher values for posterior HOXC genes (HOXC10-HOXC13) (**Figures 4D, E**). Higher methylation patterns of HOXC antisense RNA HOXC-AS1, HOXC-AS2, and the microRNA MIR-196a-2 located within the posterior region in MSCs could additionally point toward a differential regulation of respective HOX genes here. Within the HOXD gene regions, a similar methylation pattern could be detected for HOXD1 and the HOXD antisense growth associated lncRNA HAGLR/HOXD-AS1, and a decreased methylation pattern for HOXD3 and HOXD4 in FIB (**Figure 4F**). Increased miR-10b and HOXD-AS2 methylation values potentially impacted more central HOX genes and the HOXD4 and HOXD5 candidates were detected in MSCs. According to the higher and similar gene expression levels of HOXD8, respective gene sites were rather unmethylated in both cell types. In contrast to the increased expression levels of posterior HOXD genes in FIB, increased methylation values were detected in those regions (**Figure 4F**). To further validate the microarray data and in order to establish a potential HOX code for FIB, expression levels of the 39 known human HOX genes were quantified in FIB in comparison to MSCs by quantitative Real-Time RT-PCR (**Figures 5A–D**). The HOX genes HOXA4, HOXA6, and HOXA7; HOXA9, HOXB5, and HOXB7; HOXC6, HOXC8, and HOXC9, as well as HOXD8, were found to be highly expressed in both mesodermal cell types with HOXA6 being further significantly upregulated in MSCs, together with HOXB5 and HOXB7 by tendency. HOXC6 and HOXC8 were again significantly upregulated in MSCs. Significantly upregulated HOXA3 expression levels were further prominent in MSCs compared to rather low levels in FIB. In contrast, a differential expression particularly of HOXA11, and HOXA13, and to a lesser extent of HOXB9 but again of HOXB13, HOXC12, and HOXC13 by tendency, as well as again particularly of HOXD1, HOXD4, HOXD9, HOXD19, and HOXD13 were found to be upregulated in FIB compared to MSCs with generally low to very low expression levels in MSCs. The differential HOXD10 expression (significantly increased in the FIB) was especially noticeable here. In a respective methylation quantification of the CpG sites at the subregions, including the transcriptional initiation sites TSS1500 and TSS200, the 5′ untranslated region (5′UTR) between the TSS and the ATG start site and 3′UTR between the stop codon and poly-A tail (**Figures 5E–H**) further highlighted increased methylation values of anterior HOXA gene regions, particularly of HOXA3 and of HOXA-AS2 and HOXA-AS3 in FIB as well as increased methylation values of posterior HOXA gene regions, particularly of HOXA10 together with HOXA-AS4 and MIR-196b in MSCs. A comparison of the expression profiles of respective gene sets differentially regulated in MSCs versus FIB with the methylation profiles of both cell types showed a very good match (**Figure 5I**) that might account for the ‘biomarker potential’ of the respective HOX genes. To further reveal potential differences in methylation value distributions between genomic HOX regions, we determined methylation levels according to the region functional categories (**Figure 6**). Concerning the HOXA region, all CpG sites in the upstream of the transcription start site TSS1500, TTS200, 5′ UTR, and in the first exon as well as in gene body and 3′ UTR were highly methylated in FIB compared to MSCs, whereas no difference could be observed for the HOXB and HOXC regions (**Figure 6A**). In the HOXD region, higher methylation levels only in TSS1500 were detected for FIB. According to the differences in methylation pattern, particularly for the HOXA and HOXD regions, and the estimated differential expression levels for the HOXA and HOXD candidate factors (increased expression levels for HOXA3 and HOXA6 in MSCs and increased HOXA11, HOXD1, HOXD10, and HOXD13 expression levels in FIB) as stated before, we closely examined the methylation value distributions in these HOX genes (**Figure 6B**). Increased overall methylation values were detected for the HOXA3 gene in FIB, particularly in the 5’ UTR and protein-coding (gene body) regions. In MSCs, the rather low methylation values were distributed within all the indicated HOXA3 regions. Similarly, for the HOXA6 gene, high methylation values were estimated for 5’ UTR and protein-coding regions in FIB with low methylation sites in non-coding regions, and (again) there were low overall methylation levels in these regions in MSCs. In contrast, lower methylation patterns were found in the HOXA11 protein-coding regions and 3’UTR in FIB. No obvious differences became prominent in the methylation pattern of the HOXD1 gene in FIB compared to MSCs. This was even so for the HOXD10 and HOXD13 genes, indicating that the reported differential gene expression of the HOXD candidate genes might be more regulated by MIR-10b and HOXD-AS2, both of which were found to be highly methylated in MSCs. Overall, DNA methylation was more prevalent within gene bodies and 3’ UTR than in promoter regions in both cell-types with even increased methylation patterns in both region categories in FIB (**Figure 7A**).

In order to evaluate if methylation patterns could alternatively and/or additionally be used to define the MSC or FIB identity of highly similar mesodermal cells, we closely examined the methylation levels of the differentially methylated regions that were highly methylated in MSCs compared to FIB (**Figures 7B, D**). According to the ‘Epi-MSC Score’ (45) using the four candidate CpGs serpin peptidase inhibitor B5 (SERPINB5: cg00226904), chromosome 3 open reading frame 35 (C3orf35: cg22286764), cell death-inducing DFFA like effector C (CIDEC: cg05684195), and adipocyte-specific adhesion molecule (ASAM: cg19096475) it was validated that MSCs can be discriminated from FIB (**Figure 7B**). The highest difference in mean DNA methylation in MSCs compared to FIB further identified zinc finger transcription factor Casz1, WNT10A, the vestigial-like family member VGLL4, and particularly HOXA3/HOXA-AS3 CpGs as being highly methylated in FIB and T-Box transcription factor 15, SNX29, and adenylyl cyclase 9 (among others) CpGs being highly methylated in MSCs (**Figures 7C, D**).

### 3.3 ‘Superior’ regenerative potential of MSCs

**Figure 8:**
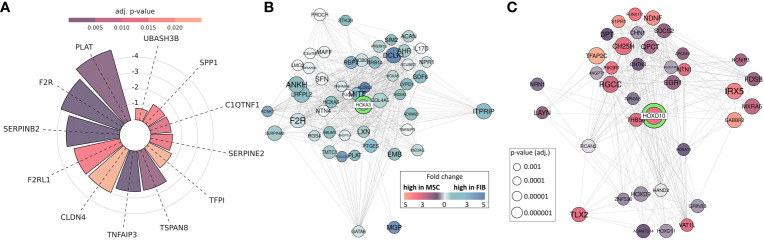
MSC-enriched genes that are of central importance in promoting successful tissue regeneration. **(A)** Differentially expressed genes between MSC and FIB enriched in GOBP ‘Regulation Of Wound Healing’ and ‘Regulation Of Response To Wound Healing’. Respective lengths of the bars denote the fold change between MSC and Fib, colors display the adjusted p-value. **(B, C)** Differentially expressed genes network analysis: Differentially expressed genes associated with HOXA3 and HOXD10. All genes with significantly (adj. p < 0.001; limmas moderated t-test, adjusted by “BH”)) higher expression in FIB, which are associated in curated gene sets with HOXD10 are shown **(B)**, and all genes with significantly (adj. p < 0.001) higher expression in MSCs, which are associated in curated gene sets with HOXA3 are shown **(C)**. The size of the gene circle depicts the adjusted p-value of this gene and the color depicts the fold change between FIB and MSCs.

Thus, epigenetic signatures together with global gene expression profiles, particularly concerning the cell-type specific HOX code, provide a powerful tool to discriminate MSCs from phenotypically similar cells. To finally highlight the superior functional properties of MSCs with respect to their regenerative capacity, and to establish a potential link between the reported HOX gene candidates, we investigated genes found to be overexpressed in MSCs and constitute the gene ontology terms ‘Wound Healing’ as determined following GSEA (**Figure 8A**). Among the potentially “regenerative” genes specifically expressed by MSCs, the fibrinolytic enzyme tissue-type plasminogen activator (PLAT), a secreted serine protease that converts the proenzyme plasminogen to plasmin, was identified together with the serine proteinase inhibitor SERPINB2, also termed plasminogen activator inhibitor-2, known to be associated with the regulation of various stem cell functions as well as protease-activated receptor 1 F2R/PAR1 and F2RL1/PAR2 with the ability to mediate non-hemostatic effects. Notably, most of the genes appeared in the gene-gene interaction networks when analyzing the potential interaction of differentially expressed HOX genes, particularly concerning the increased expression of HOXA3 in MSCs (**Figure 8B**). Concerning the increased expression of HOXD cluster genes in FIB, particularly HOXD10, an association with other HOXD and all posterior HOX genes could be revealed, namely HOXD9, HOXD13, and HOXA11 (**Figure 8C**). Among the HOXD10 interaction partners, other important genes for extracellular matrix remodeling, e.g., HAND2 (heart and neural crest derivatives expressed 2), together with known fibroblasts genes, e.g., RCAN2 (calcineurin 2) and EGR1 (early growth response gene 1) were found.

## 4 Discussion

Consistent with the initial description as ‘fibroblast precursor’, there are no obvious molecular markers to clearly distinguish MSCs from other mesenchymal cells, especially fibroblasts. Generally, MSCs show a fibroblast-like morphology and are, like fibroblasts, defined as non-endothelial, non-epithelial, and non-hematopoietic adherent cells of mesenchymal origin. Thus, fibroblasts and MSCs share highly similar mesenchymal phenotypes and even to a certain extent their gene expression profiles, raising the question of whether MSCs and fibroblasts really represent different cell types. It has already been suggested that both cell types are the same and that fibroblasts could represent aged MSCs (46). In combination with the fact that most MSC sources require invasive extraction means compared to fibroblasts being harvested more easily in large numbers from various biological wastes, the use of fibroblasts has even been suggested as a practical alternative to MSCs (47). Single-cell transcriptome integration analysis using MSCs of various sources (umbilical cord, foreskin, bone marrow, and adipose tissue) then suggested that MSCs represent a subclass of fibroblasts, as distinct cell subsets identified within the MSC populations also showed a fibroblast phenotype but subsets with the fibroblast phenotype did not necessarily show the MSC phenotype (48). Here, we used gene expression and methylation analysis to provide (i) further evidence that MSCs and particularly VW-MSCs can be discriminated from phenotypically similar fibroblasts based on a cell type-specific gene code (the ‘HOX code’), thus representing different cell types and that (ii) based on these difference in profiles, additional superior repair capabilities could be estimated for (VW-)MSCs.

### 4.1 The HOX code of fibroblasts as compared to MSCs

**Figure 9:**
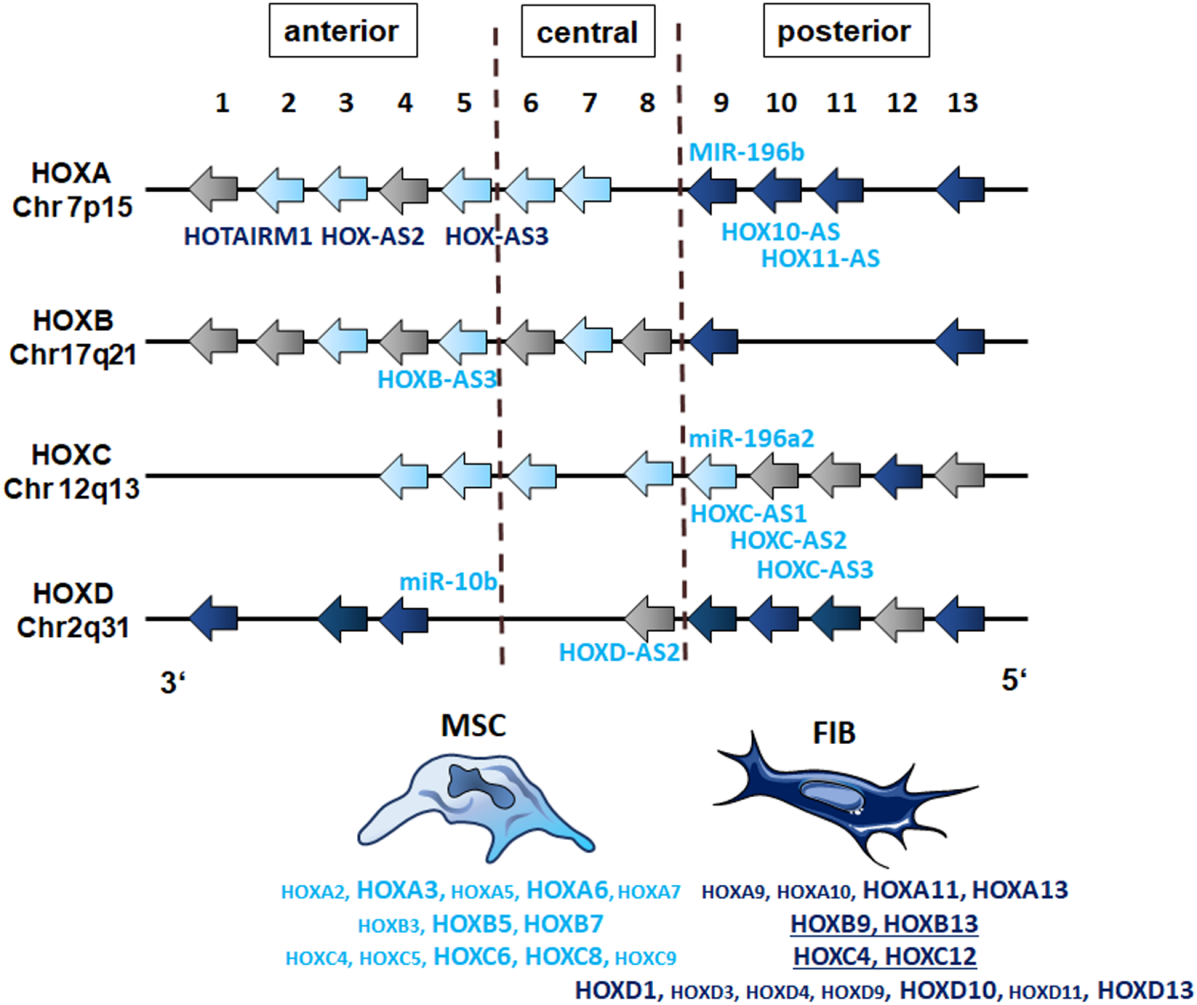
The HOX code of FIB as compared to MSCs. Based on the genome-wide gene expression and methylation analyses, patterns of HOX gene expression (‘the HOX code’) as determined in vascular wall-derived MSCs compared to dermal fibroblasts are schematically summarized. Differentially expressed HOX genes as determined by the microarray analyses of MSC are colored in light blue and of FIB in dark blue. Validation of differentially expressions by qRT-PCR is indicated by enlarged capitals. Additional, differentially expressed candidates as determined by qRT-PCR are underlined (FIB). The position of non-coding RNAs that are interspersed within the coding HOX genes are listed (MIR, microRNAs; AS, antisense RNAs). The light blue color indicates high methylation values for MSCs and the dark blue color indicates high methylation values for FIB as estimated by the methylation analysis.

GSEA using MSC and fibroblast-specific gene sets confirmed the respective identity of each cell type, and at the same time successfully discriminated both cell types from each other. The primary dermal fibroblasts used in the present study could be ‘verified’ as fibroblasts using a specified set of fibroblast-enriched transcription factors as potential cell identity genes maintaining the fibroblast state (41, 43). The differential expression of homeotic genes, particularly HOX genes encoding transcription factor proteins that contain a region called the homeodomain and represent master regulator genes in terms of cellular identity, was further investigated and specified, finally yielding a fibroblast-specific HOX code (**Figure 9**). Global gene expression profiling and qRT-PCR validation in combination with the respective gene methylation analysis revealed a potential HOX code for (dermal) fibroblasts comprising particularly the posterior HOX genes HOXA9, HOXA11, and HOXA13, as well as HOXD1, HOXD3, HOXD4, HOXD9, HOXD10, and HOXD13 (**Figure 9**). Additionally, HOXB9, HOXB13, and HOXC12 together with HOXD3, HOXD4, and HOXD9 could be suggested as additional but minor HOX code candidates in FIB. As revealed previously, and confirmed in the present study (16, 21, 27, 44), mainly central HOX genes and HOX genes neighboring closely to the central cluster region were shown to be expressed within all the different tissue-specific MSCs, and particularly increased expression levels of HOXB7, HOXC6, and HOXC8 but also of HOXA3 and HOXA7, and can be detected in VW-MSCs (**Figure 9**). The direct MSC-fibroblast comparison presented in this study further revealed the prominent role of HOXA3 in VW-MSC, especially concerning their therapeutic potential.

In investigating the HOX expression profiles of (synovial) fibroblasts according to the positional identity, it was already revealed that posterior HOXA (especially HOXA13) and HOXD genes (HOXD10, HOXD11, and HOXD13) could be found in fibroblasts from distal origins (49). HOXA13, particularly, was shown to maintain a distal-specific transcriptional program in adult fibroblasts, and HOXD gene expression was limited to dermal fibroblasts. Similarly, HOXC13 expression could be attributed to fibroblasts derived from the skin. Recently, high expression levels of HOXA7, HOXA9, HOXC8, and HOXC11 were found to be expressed in healthy skin fibroblasts with HOXA7, HOXA9, and HOXC8 being significantly downregulated in the respective fibroblasts of fibroproliferative lesions (50). Thus, fibroblasts were shown to exhibit site-specific differences in HOX gene expression that were related to their positional identity. At the same time, the HOX code was maintained upon culture, strongly suggesting that the fibroblast-specific HOX code has (i) a role in the establishment of site-specific fibroblast gene expression program (51), and (ii) may be maintained via epigenetic regulation (52). HOXA9 and HOXC10, for example, were reported to be upregulated in highly proliferating fibroblasts, finally forming hypertrophic scars (53). Similar to the role of HOXA9 in regulating fibroblast proliferation, HOXB9 overexpression was shown to be involved in excessive proliferation, finally facilitating hypertrophic scar formation (54).

Concerning fibroblast proliferation and extracellular matrix remodeling, the central role of the posterior HOXA gene HOXA11 and the HOXD gene HOXD10 has become prominent in the literature and is further highlighted by the results presented here. HOXA11 levels in fibroblasts were shown to regulate fibroblast proliferation as well as the expression of collagens and matrix metalloproteinases (55). At the same time, HOXA11 was shown to be higher expressed compared to MSCs, correlating with the higher methylation pattern of the HOXA11 gene in MSCs. Studies using synovial lining fibroblasts (also called fibroblast-like synoviocytes) further revealed that HOXD10 expression is of critical importance for maintaining a quiescent fibroblast state as diseased fibroblast states (in rheumatoid arthritis), characterized by abnormal HOXD10 levels, impacted on increased cellular viabilities as well as abnormal migration via increased p38/c-Jun N-terminal kinase p38/JNK signaling (56). Likewise, although not estimated in fibroblasts, HOXD10 was shown to maintain a quiescent, differentiated phenotype (in endothelial cells) by suppressing cell migration and the expression of genes involved in extracellular matrix remodeling, e.g., matrix metalloproteinases and particularly the plasminogen activator receptor (57). Thus, HOXD10 might function as a general mediator of cellular quiescence. Analyses of potential HOXD10 interaction partners further revealed an association with other posterior HOX genes, especially HOXA11, HOXD9, and HOXD13 as well as genes important for extracellular matrix remodeling as potential fibroblast-specific features. For example, HAND2 overexpression was particularly shown to mediate fibroblast marker expression and regulate extracellular matrix organization and function by maintaining the balance between integrins and fibronectin (58, 59). Likewise, EGR1, a known connective tissue gene regulating extracellular matrix deposition and organization, was found to be differentially expressed in fibroblasts and potentially linked to differentially expressed HOXD10 here (60). Furthermore, EGR1 expression might be of central importance for maintaining a certain mesenchymal phenotype as EGR1 regulates the expression of epithelial transcriptional inhibitors (e.g., SNAIL and SLUG) via the RAF–MEK–ERK pathway, and thus regulates epithelial-mesenchymal transition (61).

In MSCs, it is already known that besides the central role of HOX genes in defining cellular identity, expression levels and the activity of HOX genes decisively impact their respective cellular functions. The VW-MSC specific expression pattern of the *HOX* genes *HOXB7*, *HOXC6*, and *HOXC8* turned out to be one characteristic to discriminate VW-MSCs from phenotypically similar cells like fibroblasts. These MSCs, as well as *in vitro*-generated vascular wall-typical MSCs, were shown to preferentially differentiate into mural cells, namely pericytes and smooth muscle cells (21, 27, 62, 63). Likewise, the colony-forming capacities and differentiation potentials into chondrocytes, osteocytes, and adipocytes turned out to be important properties to discriminate MSCs from fibroblasts and thus could be associated with the differential expression of HOXB7, HOXC6, and HOXC8 in VW-MSCs (21, 24, 25). MSCs, and especially artery-wall-derived MSCs, were furthermore reported to be superior over fibroblasts in terms of anti-inflammatory and wound healing repair features (21, 24). Accordingly, it has been suggested that MSCs might be fibroblasts with beneficial properties (64). The increased expression of central HOX candidates herein might reflect the beneficial functional properties with activation and maintenance of HOX gene expression generally being not only auto- and cross-regulated by respective HOX proteins themselves but also by long noncoding RNAs (lncRNAs) that can interact with Polycomb and Trithorax group complexes to modulate transcription and chromatin conformation and thus modulate Hox gene expression (65). Expression of the lncRNA HOTAIRM1 (HOXA transcript antisense RNA myeloid 1), known to be involved in transcriptional regulation of the HOXA genes in embryonic and even in cancer stem cells, was shown to be decisive for their proliferation and self-renewal together with the HOTAIRM1 neighboring genes, HOXA1, HOXA2, and HOXA3 (66). Especially, HOXA3 was found to be highly expressed in the VW-MSCs. HOTAIRM1 was shown to be highly expressed in adult stem cells and especially in MSCs promoting osteogenesis of MSCs (via JNK/AP-1 signaling) and chondrogenesis (via JNK/MAPK/ERK pathway). (67, 68). LncRNA HOXA-AS2 was further identified as a positive regulator for the osteogenesis of MSCs through inactivating NF-κB signaling (69), and aberrant upregulation of lncRNA HOXA-AS2 was even suggested to promote regulatory T cell proliferation and immune tolerance (70). Another lncRNA, HoxA-AS3, was shown to regulate lineage commitment of MSCs by interacting with histone methyl transferase EZH2 that was generally associated with H3K27 methylation and gene silencing (71). HOXA-AS3 expression was important for adipogenic differentiation of MSCs while a knockdown of HOXA-AS3 expression in MSCs exhibited an enhanced osteogenesis potential. Mechanistically, HOXA-AS3 EZH interaction was required for H3K27me3 of key osteogenic transcription factor Runx2 (71). Upregulated HOXA-AS3 expression levels furthermore were shown to foster cell proliferation and migration while inhibiting apoptosis (72). Notably, in the present study, increased methylation patterns for these lncRNAs could be revealed in fibroblasts.

With regard to the posterior HOX regions, HOXA10-AS, also known as HOXA cluster antisense RNA 4, turned out to be decisively involved in stem cell proliferation while inhibiting apoptosis by activating HOXA10 expression (73). Likewise, HOXA11-AS, as a known co-activator of the HOXA genes, was shown to regulate HOXA11 mRNA by blocking transcription. Accordingly, both genes were found to be highly methylated in VW-MSCs while the respective HOXA10 and HOXA11 expression levels were hardly detectable in these cells.

Similar to the small non-coding RNAs, three Hox cluster microRNAs (posttranscriptional regulators) genes that generally bind to the 3′ UTRs of messenger RNAs, thereby regulating mRNA stability or translation, are known: MIR-196b, located between HOXA9 and HOXA10, MIR-196a-1 in the intergenic region between HOXB9 and HOXB10, and MIR-196a-2 located between HOXC9 and HOXC10 (74). MIR196b, for example, was found to be highly methylated in our MSCs, together with clearly reduced HOXA5 and HOXB8 transcript levels, in line with the findings that MIR-196b mediated downregulation of HOXA5 (and HOXB6) transcripts (75) and even repression of HOXB8. Concerning the HOXB cluster region, HOXB5 and HOXB7 were found to be overexpressed in MSCs, which fits with the observation that HOXB7 expression is important to activate RUNX2 as an osteogenic differentiation inductor and thus promoting osteogenesis (76). Besides, MIR-10b is encoded in the HOXD locus neighboring HOXD3 and HOXD4, and a knockdown of HOXD-AS2 was shown to reduce the expression of MIR-10b and HOXD genes (77). Increased MIR-10b methylation could be observed in MSCs, in parallel to the reduced expression of early and, indeed, all HOXD genes compared to high expression levels in fibroblasts.

In summary, the cell type-specific HOX gene code presented here together with the cell-type specific features (of MSCs) could be used to identify certain lineage cells among phenotypically similar cells. Particularly, the expression of HOXA and HOXD candidate genes, namely increased expression levels for HOXA3 and HOXA6 in MSCs and increased HOXA11, HOXD1, HOXD10, and HOXD13 expression levels in FIB could serve as additional cell-type specific marker genes.

### 4.2 Epigenetic classification of MSCs compared to fibroblasts

HOX gene expression levels of the HOXA and HOXD candidate factors matched convincingly with the methylation profile of the respective genes. Increased methylation patterns for the TSS1500, TSS200, 5’ UTR, 1^st^ exon, gene body, and 3’ UTR of the HOXA cluster region were generally detected in fibroblasts together with a slight but not significant increase in the respective sites of the HOXD cluster region. Concerning the additional HOX code factors for each cell type, reduced methylation values were detected for the HOXA3 gene in MSCs, particularly in the gene body regions. The HOXA6 gene showed a similar methylation pattern. In contrast, the fibroblast HOX code factor HOXA11 turned out to be more methylated in the protein-coding region in MSCs. No obvious differences became prominent in the methylation pattern of the HOXD1 gene in FIB compared to MSCs. This was even so in the HOXD10 and HOXD13 genes, indicating that the reported differential gene expression of the HOXD candidate genes might be more regulated by MIR-10b and HOXD-AS2, both of which were found to be highly methylated in MSCs. Thus, at least concerning the HOXA genes and concerning the overall DNA methylation profile, methylation was more prevalent within gene bodies than for promoters or generally upstream gene regions, and gene-body methylation was observed to be positively correlated with gene expression levels. This is in line with recent findings, that although methylation in gene promoters was generally associated with transcriptional silencing, differential methylation of regions downstream of the transcription start site was found to be more informative for the respective gene expression (78, 79). Moreover, an inverse correlation between promoter methylation within CpG islands and gene expression data was observed in numerous human tissue specimens, thus suggesting that (permanently) methylated CpG sites located predominantly in gene-body regions are mediators of tissue-specific gene regulatory mechanisms and thus decisive for regulating active transcription (80, 81).

As the expression of positional HOX determinants during development and the respective maintenance in adulthood is most likely due to epigenetic mechanisms on the one hand (82), and epigenetic signatures, i.e., methylation patterns of phenotypical similar cells, are suggested to reveal a usable promising difference between them on the other hand (46, 83), we additionally investigated differentially methylated regions that were highly methylated in MSCs compared to FIB. With the already reported epigenetic classification of different MSC types compared to fibroblasts finally providing a fast, cost-effective, and transparent classification of MSCs that successfully discerns MSCs from fibroblasts, we confirmed and validated the methylation differences in the CpGs SERPINB5, C3orf35, CIDEC, and ASAM, known as the ‘Epi-MSC Score’ (45) for the primary VW-MSCs and dermal fibroblasts used in the present study. Besides the highest difference in mean DNA methylation of particularly HOXA3/HOXA-AS3 CpGs in MSCs versus FIB as described above, we identified that WNT10A and the vestigial-like family member VGLL4 -among others-, were highly methylated in fibroblasts, and thus could account for stem cell-specific properties in MSCs. According to the general findings that canonical Wnt signaling is important for stem cell proliferation while noncanonical WNT signaling appears to induce cellular senescence, the canonical WNT ligand WNT10A was shown to regulate the proliferative capacity of (synovial) MSCs (while limiting senescent cell states) (84, 85). Lower methylation patterns in MSCs were also detected for the VGLL4 gene, a co-transcriptional regulator of stem cell survival while fostering the colony formation capacity via interaction with the Rho/Rock pathway (86). In contrast, higher methylation of the T-Box transcription factor 15 in MSCs together with the generally more repressive role of T-box domain transcription factors could resemble a fibroblast-specific phenomenon as lower methylation values of the TBX15 gene might account for increased transcriptionally active TBX15 preventing fibroblasts from a myoblast phenotype (87, 88).

### 4.3 Additional factors promoting the therapeutic potential of MSCs

Although MSCs and FIB seem almost identical with respect to their surface immunophenotype and at least to a certain degree concerning their gene expression profiles, differences in the methylation as well as gene expression profiles together with differences in clonogenicity and differentiation capacities could successfully be used to distinguish both cell types and further for in-depth characterization of both cells types. That also includes the putative superior immunomodulatory capacities of MSCs making MSCs more efficient than fibroblasts.

Inflammation-driven migration, for example, seems to be a superior cellular feature of MSCs as endometrial MSCs showed higher migration activities compared to fibroblasts isolated from the same organ, and the migration capacity decreased when MSCs differentiated into these cells (89). Accordingly, several cytokines were preferentially secreted by endometrial MSCs compared to fibroblasts isolated from the same organ, including vascular endothelial growth factor-A, stromal cell-derived factor-1 alpha, interleukin-1 receptor antagonist (IL-1RA), IL-6 and IL-8, monocyte chemoattractant protein-1 (CCL2), macrophage inflammatory protein 1α, and RANTES (89). In a more general approach, a genome-wide transcriptome comparison of dermal fibroblast populations with MSCs corresponding to five tissue origins (bone marrow, fat, amnion, chorion, and cord) showed a clear segmentation between skin fibroblasts and all MSC samples (90). Gene ontology term analysis in order to determine biological characteristics distinguishing the fibroblast and MSC groups revealed terms and particularly transcripts related to the structuration of the tissue skeleton including numerous ECM, focal adhesion, and the cyto- and nucleoskeletons, as well as secreted factor transcripts that were upregulated in fibroblasts, finally suggesting a signature of 42 candidates directly related to the structuration and composition of the ECM network, representing biological differences between both cell types (90). The molecular signature of fibroblasts included the overexpression of transcripts related to the collagen meshwork, such as genes important for collagen processing together with collagen fibril anchorage points, as well as elastic network transcripts (90). Accordingly, it was suggested that fibroblasts are stromal cells that provide more of the structural framework for almost all tissue types, whereas MSCs account more for the (age-dependent) regenerative capacity (91). However, these phenotypically similar cells, being similarly localized, act closely together in response to tissue injury. In (dermal) wound healing processes, for example, recruited (and activated) MSCs ‘animate’ fibroblasts to produce the appropriate amounts of collagen fibers needed for tissue repair. MSC-secreted factors at the same time prevent scar formation and limit inflammation, fostering a return to a normal healthy state (92).

Concerning their regenerative potential, we could additionally identify and highlight the factors that underline the superior capacities of MSCs in terms of ‘healing’, including the plasminogen activator system of which the tissue plasminogen activator (tPA, PLAT) was highly differentially expressed in MSCs compared to fibroblasts. tPA (like urokinase plasminogen activator) converts plasminogen to the active enzyme plasmin, a trypsin-like protease, finally resulting in fibrin clots degradation forming within blood vessels. Genetically modified MSCs overexpressing tPA have already been suggested as a therapeutic strategy for thrombolytic therapy in which the forced expression and secretion of tPA in MSCs together with their prominent characteristics of low immunogenicity and homing towards damaged tissue sites could be used to achieve lesion-targeting medication (93). As tPA itself has been established as a thrombolytic agent to be used as the first line of treatment of acute myocardial infarction (94), and high tPA expression and secretion via extracellular vesicles by MSCs have been found to be beneficial, particularly for stroke therapy (95), MSC-mediated tissue repair, especially targeted treatment of a thrombus using MSC-delivered tPA, seems to be a promising option. Of note, plasminogen activators (together with MMP-14, CCL2, and CXCL12) were already identified as downstream HOXA3 target genes that are important for regulating tissue integrity and limiting the expression of inflammatory mediators (96). Here, we identified the plasminogen activator system as an MSC-specific property as MSCs were characterized by a differential and increased expression of HOXA3 (compared to fibroblasts). The shown differential expression of HOXA3 in our MSCs together with the fact that the tissue plasminogen activator was already identified as a HOXA3 downstream target, as well as the differential expression of HOXD10 in fibroblasts known to suppress the plasminogen activator system, clearly indicate a regulation of cell type-specific function by the HOX code, presumably the superior regenerative capacities of MSCs. Other stem cell-specific features, such as colony forming and differentiation potential, were already linked to a differential HOX gene expression (21, 24, 25).

Besides the significant role of the plasminogen activator system in fibrin clot surveillance, the system also contributes to extracellular proteolysis in physiological (e.g., tissue remodeling, cell migration, wound healing, and angiogenesis) or pathological (e.g., acute and chronic inflammation, preeclampsia, tumor invasion, and metastasis) processes (97). In addition to fibrin degradation, plasmin can degrade several ECM and adhesion proteins (e.g., proteoglycans, laminin, and fibronectin) and even activate pro-collagenases that in turn foster collagen degradation. tPA, together with the protease-activated receptor-1 (F2R) were even shown to be involved in endothelial cell proliferation and survival, when (chorionic villous) MSCs protected endothelial cells from injury induced by high levels of glucose (98). F2R, also known as coagulation factor II thrombin receptor or proteinase-activated receptor 1 (PAR1), was even shown to impact adipogenesis, especially when MSCs differentiate into adipocytes, suggesting F2R as a reliable adipogenic marker (99). Concerning the differentiation potential of MSCs into osteoblasts and adipocytes, an inverse relationship between the two cell types became prominent. Accordingly, F2RL1 (F2R Like Trypsin Receptor 1), also known as coagulation factor II (thrombin) receptor-like 1 or proteinase-activated receptor 2 (PAR2) was shown to regulate osteoblastic MSC differentiating while inhibiting adipogenesis (100).

Thus, differences in both cell types, particularly concerning the differentiation capacities in MSCs, became prominent together with increased regenerative potential. In line with the assumption that MSCs exhibit putatively superior immunomodulatory capacities, we could additionally specify increased serine protease inhibitor (SERPIN) expression levels in MSCs, namely SERPINB2 (Serpin Family B Member 2), which was even present in the interaction nodules of HOXA3. Serine protease inhibitor expression by MSCs was shown to mediate host immune response evasion as the basis for the use of allogeneic MSCs (101). MSCs are generally considered to be hypoimmunogenic cells because these cells lack expressions of major histocompatibility complex class II molecules as well as of classical positive costimulatory molecules, which would ultimately be necessary for the activation of CD4 and CD8 T cell responses. Likewise, MSCs escape from the granzyme B lytic activity of CTLs, as granzyme B activity is tightly regulated through its interaction with peptidase inhibitors of the SERPIN superfamily that are shown to be expressed in MSCs (102). For clinical applications, a very good and sustained viability of MSCs is highly desired to increase therapeutic efficiency. Therefore, enhancing the expression of SERPINs may be a useful strategy for the potentiation of MSC defense strategies, particularly for increasing the ability of MSCs to escape NK cell-mediated lysis.(103). In addition, SERPINB2, which was first identified as a placental tissue-derived uPA inhibitor and called plasminogen activator inhibitor type 2 (PAI-2) (104), was shown to be an essential factor for the osteoblastic differentiation potential of MSCs (105).

## 5 Conclusion

Transcriptomic profiling including single-cells and respective methylation analyses specified the molecular portraits of mesodermal cells, particularly of MSCs and fibroblasts, which is not only proof of identification and differentiation of phenotypically highly similar cells, it further significantly improved cell class definitions and can serve as a guide for further studies. Our present study contributes to this molecular and epigenetic characterization by analyzing an additional type of MSCs, namely vascular wall-derived MSCs. Thereby, we specified the already known VW-MSC-specific cell identity code, namely the ‘HOX code’ comprising the HOX genes HOXB7, HOXC6, and HOXC8 that were already linked to classical stem cell features, particularly multipotency and clonogenicity, by identifying HOXA3 as an additional HOX candidate factor. The differential expression of HOXA3 in MSCs and matching methylation patterns could be associated with the superior regenerative capacity of MSCs, especially concerning their immunosuppressive action, which is a prerequisite for the clinical application of MSC-based therapies. Accordingly, the established HOX code for fibroblasts revealed a central role of posterior HOX genes for fibroblast identity and for fibroblast-specific function, highlighting HOXA11 and HOXD10 as potentially central fibroblast cell biomarkers that are responsible for extracellular matrix remodeling features, while generally mediating fibroblast quiescence. Thus, in-depth analyses of cell type specific gene codes either based on expression and/or methylation profiles together with cell-type specific features can not only be successfully used to identify and characterize certain lineage cells among phenotypical similar cells, they will also pave the way for manipulating these cells either in diseased states or for improving certain cellular features, e.g., for therapeutic benefits.

## Data availability statement

The datasets presented in this study can be found in online repositories. The names of the repository/repositories and accession number(s) can be found below: GSE240685- DNA methylation and GSE240831-gene expression (GEO).

## Ethics statement

The studies involving humans were approved by Ethik-Kommission, University Medical Faculty, Essen. The studies were conducted in accordance with the local legislation and institutional requirements. Written informed consent for participation in this study was provided by the participants’ legal guardians/next of kin.

## Author contributions

BB: Data curation, Formal Analysis, Investigation, Visualization, Writing – review & editing. KU: Data curation, Formal Analysis, Software, Writing – review & editing. JH: Data curation, Formal Analysis, Writing – review & editing. HS: Data curation, Formal Analysis, Writing – review & editing. DK: Conceptualization, Data curation, Formal Analysis, Funding acquisition, Methodology, Project administration, Resources, Supervision, Validation, Visualization, Writing – original draft, Writing – review & editing.
